# Predicted Concentrations and Optical Properties of Brown Carbon from Biomass Burning over Europe

**DOI:** 10.1021/acsestair.4c00032

**Published:** 2024-05-28

**Authors:** Ksakousti Skyllakou, Marios-Bruno Korras-Carraca, Christos Matsoukas, Nikos Hatzianastassiou, Spyros N. Pandis, Athanasios Nenes

**Affiliations:** † Institute of Chemical Engineering Sciences, ICEHT/FORTH, Patras 26504, Greece; ‡ Department of Environment, University of the Aegean, Mytilene 81100, Greece; § Laboratory of Meteorology and Climatology, Department of Physics, University of Ioannina, Ioannina 45110, Greece; ∥ Department of Chemical Engineering, University of Patras, Patras 26504, Greece; ⊥ Laboratory of Atmospheric Processes and their Impacts, École Polytechnique Fédérale de Lausanne, 1015 Lausanne, Switzerland

**Keywords:** aerosol optical depth, single scattering albedo, direct radiative forcing, wildfires, absorption

## Abstract

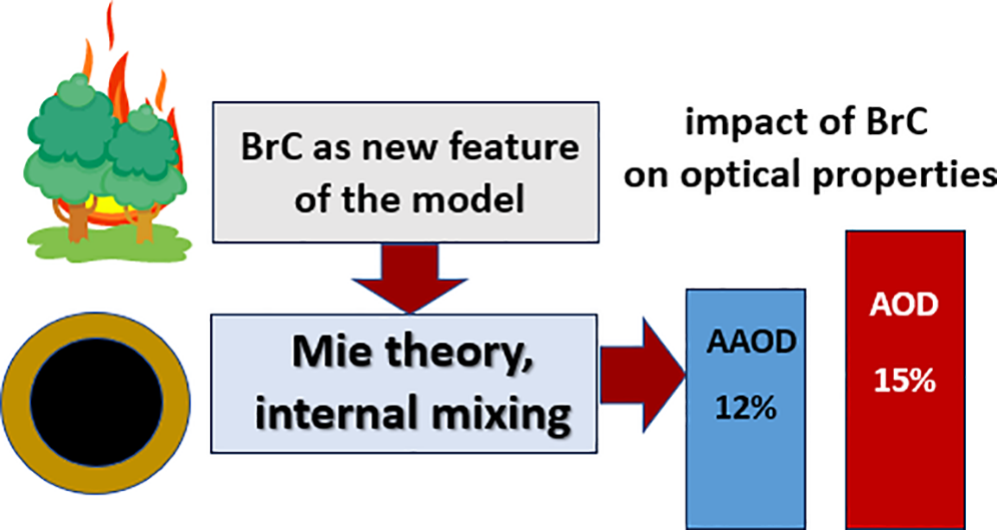

Black carbon (BC) and brown carbon (BrC) are light-absorbing carbonaceous aerosol components that can contribute to radiative forcing and thus affect the climate. In this study, we focus on the modification of aerosol optical properties associated with BrC emissions from biomass burning. BrC is simulated with the introduction of three new species in the three-dimensional chemical transport model PMCAMx-SR, two primary-absorbing (inert and reactive BrC) species, and one “photobleached” BrC species. 10% of the emitted BrC is assumed to be inert, and the rest to be reactive and able to undergo photobleaching. The scattering and absorption coefficients of the aerosol for different wavelengths are estimated by using Mie theory. BrC causes a 5–15% increase of the aerosol optical depth at 550 nm in regions affected by fires and can increase light absorption by up to 12% compared to when there is no BrC. During major biomass burning events, the absorption of BrC can reach up to 13% of that of BC at 550 nm and 25% at 440 nm. The ability of the model to reproduce measured absorption is improved when BrC is added to the light-absorbing components.

## Abstract 2

This study focuses on the simulation of brown carbon emitted from wildfires and quantifies its impact on aerosol optical properties.

## 1 Introduction

The absorbing part of organic aerosol (OA) in the visible and ultraviolet (UV) range is known as BrC.^1,2^ The main primary sources of BrC in the atmosphere are biomass combustion^2−7^ and secondary production during the oxidation of emissions of the combustion of biofuels and fossil fuels.^8^ Also, the secondary production of BrC has been observed in aqueous phase reactions often accompanied by the in-cloud formation of humic-like substances (HULIS)^2^ and during heterogeneous reactions of polycyclic aromatic hydrocarbons (PAHs) with nitrate radicals.^9^ In this study, we will focus on the dominant source of BrC, wildfires. Wildfires are a significant source of OA globally, in Europe^10−14^ and even in the arctic.^15^

The evolution of BrC in the atmosphere includes changes in its light absorption properties through photobleaching. Photobleaching refers to the aging of the BrC chromophores via photochemistry and results in the reduction of the absorptivity of the BrC.^16−19^ The estimated lifetime of BrC has been estimated to be approximately 24,^20^ 18–30,^5^ and 15–28 h.^18^

The contribution of BrC to the aerosol Direct Radiative Forcing (DRF) is still uncertain, with studies reporting values ranging from +0.03 to +0.57 W m^–2^.^21−25^ A few modeling studies have tried to evaluate the predicted aerosol optical properties against available observations to explain better the impact of BrC. Chung et al.^26^ used observationally based estimates of the carbonaceous aerosol DRF, finding that BrC contributes about 20% of the total absorption at 550 nm globally. Feng et al.^21^ performed global simulations with the IMPACT model and concluded that the aerosol absorption optical depth (AAOD) increases by 18% at 550 nm due to strongly absorbing BrC. Lin et al.^22^ also evaluated the IMPACT model against AErosol RObotic NETwork (AERONET) retrievals, found that it overestimates the single scattering albedo (SSA) and it underestimates the AAOD over Europe. Wang et al.^23^ using the GEOS-Chem model found that the inclusion of BrC reduces the model bias in AAOD at multiple wavelengths by more than 50% for AERONET sites worldwide. Jo et al.^24^ also used GEOS-Chem for 2007 and they found that the inclusion of BrC in the model reduced the high bias of simulated SSA against AERONET observations by 23-33%. Brown et al.^27^ implemented BrC into CAM5 and performed simulations on global scale for 9 years (2003–2011). CAM5 underestimated the AERONET SSA in regions affected by biomass burning. Zhang et al.^28^ used the GEOS-Chem for Chiang Mai in Thailand during the dry season. They evaluated the performance of the model against AERONET data and concluded that the inclusion of BrC absorption reduced the model bias of AAOD by about 5–15%. They also estimated that the contribution of BrC to total carbonaceous aerosol absorption at 440 nm was 33–40%. Xu et al.^29^ modified the bulk aerosol optical scheme in CAM5.3 by including BrC absorption. They found that BrC inclusion improved (by 20%) the agreement between simulated aerosol optical properties and observations in Kanpur, India (a site affected by biomass burning). Drugé et al.^30^ used the ARPEGE-Climat global climate model and compared the predicted SSA, aerosol optical depth (AOD), and AAOD against data from AERONET and satellite data on a global scale. The implementation of BrC resulted in an improvement in the estimation of the total SSA and AAOD at 350 and 440 nm. Neyestani et al.^31^ applied the WRF-Chem model for August 2015 in the U.S. and tested the BrC representation into the model by evaluating the predictions against AERONET data. The model resulted in the best agreement with observations in terms of AAOD, when BrC was present. Konovalov et al.^32^ applied the CHIMERE regional chemical transport model for July 2016 during wildfire episodes in Siberia. They assumed different OA schemes and evaluated the model against satellite data. They concluded that the version of the model that assumed chemically reactive OA agreed better with the observed AOD values. Finally, Methymaki et al.^33^ applied the WRF-Chem model for August 2019 over Europe, focusing on the Mediterranean basin during the presence of wildfires. They concluded that the incorporation of BrC absorption generally improved the AAOD model performance against the AERONET and MERRA-2 data. A lot of uncertainties in previous studies are associated with the representation of biomass burning aerosol and its optical properties in the models. Brown et al.^34^ compared different climate models against observations and found that most models tend to overpredict the absorption of biomass burning particles. This is mainly associated with the assumed mixing states of BC and BrC with the other constituents of the particles.

There are limited number of studies so far^33^ that focus on the modeling of the optical properties of BrC on a regional scale and especially over Europe. This study puts effort into enhancing our knowledge of the impact that BrC may have on the aerosol optical properties during wildfire events. This is the first step before the calculation of DRE or DRF due to BrC modifications. To do this, we coupled the regional model PMCAMx-SR with the calculations of the aerosol optical properties with the inclusion of BrC. First, the addition of BrC into the model is described together with the predictions for BrC levels over Europe. The impact of BrC on the total aerosol optical properties is then analyzed.

## 2 Materials and Methods

### 2.1 PMCAMx-SR

PMCAMx-SR^35^ is an extended source-resolved version of PMCAMx,^36^ a three-dimensional chemical transport model that uses the framework of CAMx^37^ and simulates the processes of horizontal and vertical advection, horizontal and vertical dispersion, and wet and dry deposition. It also simulates chemistry in the gas, aqueous, and aerosol phases. The chemical mechanism used is the extended SAPRC mechanism.^37,38^ In this PMCAMx-SR version, SAPRC was extended to include 265 reactions of 114 gases and 18 radicals to accommodate the volatility basis set (VBS) species. The aerosol-size composition distribution is simulated using the sectional method with ten size bins for the diameter range from 40 nm to 40 μm. In total, PMCAMx-SR in this study simulates 60 aerosol components, both inorganic and organic. PMCAMx-SR has the advantage that it can simulate a source of emissions separately from the other sources; in this case, the separate source is wildfires.

For the simulation of OA, PMCAMx-SR uses the VBS framework.^39^ PMCAMx-SR uses the same volatility bins for anthropogenic and biogenic OA, like PMCAMx, except for wildfires. For the specific source of wildfires, the intermediate volatility organic compounds (IVOCs), semivolatile organic compounds (SVOCs), and the low volatility organic compounds (LVOCs) are described with nine volatility bins (10^–2^–10^6^ μg m^–3^ at 298 K), as in Theodoritsi and Pandis.^35^ Secondary Organic Aerosol (SOA) from anthropogenic volatile organic compounds (aSOA-v) and SOA from biogenic volatile organic compounds (bSOA-v) are represented by four volatility bins, with C* values ranging from 1 to 10^3^ μg m^–3^ at 298 K. Long-range transport OA (LRT) is assumed to be very oxidized OA and is treated in PMCAMx-SR as an inert OA species. Overall, the OA components included explicitly in PMCAMx-SR are fresh primary anthropogenic OA (POA), biomass burning primary organic aerosol (bbPOA), anthropogenic SOA (aSOA), biogenic SOA (bSOA), SOA from semi-volatile and intermediate volatility anthropogenic organic compounds (SOA-sv and SOA-iv), biomass burning secondary organic aerosol from semi-volatile and intermediate volatility organic compounds (bbSOA-sv and bbSOA-iv), and long-range transport OA. All the above OA components (except LRT and bSOA) are assumed to react with the OH radical, leading to a reduction in their volatility by one order of magnitude, a process called chemical aging. For the bbPOA, the rate constant used for the chemical aging reactions with the OH radical is the same as that used for fresh POA, 4 × 10^–11^ cm^3^ molecule^–1^ s^–1^, and for the aSOA it is assumed to be equal to 1 × 10^–11^ cm^3^ molecule^–1^ s^–1^.^40^

### 2.2 Brown Carbon Simulation

Brown carbon is simulated with the introduction of three new species in the three-dimensional PMCAMx-SR,^35^ two primary (absorbing) and one “photobleached” species. These BrC species are inert BrC (iBrC), reactive BrC (rBrC), and photobleached BrC (phBrC). The addition of BrC species does not replace the bbOA in PMCAMx-SR, as BrC is assumed to be a part of the bbOA. These species are treated like all the other biomass burning organic aerosol (bbOA) species in PMCAMx-SR. The iBrC does not react at all and is considered to be the persistent part of BrC, corresponding to the lowest volatility inside the model (nonvolatile), which corresponds to nonreactive particles of C* = 10^–2^ μg m^–3^. The other two brown carbon species (rBrC and phBrC) follow the volatility distribution of bbPOA in the range of C* = 10^–1^ to 10^6^ μg m^–3^. As a result, a small fraction of them can be in the gas phase, can react, and can be transferred back to the particulate phase according to the VBS framework.

The new process added in PMCAMx-SR is the reaction of photobleaching, which in the presence of sunlight consumes rBrC forming phBrC. The rate constant for this reaction is taken from Wong et al.,^18^ based on wildfire events in the Mediterranean, and is equal to 3.4 ×10^–5^ s^–1^, assuming first-order kinetics. Τhis rate constant corresponds to a lifetime of rBrC of about half a day on a typical summer period in the sampling site^18^ in Crete in Greece.

### 2.3 Calculation of the Aerosol Optical Properties

The aerosol optical depth (AOD) for each wavelength is calculated as the sum of all the individual layers of AODs:
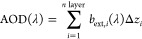
1where *b*_ext,*i*_(λ) is the extinction coefficient of layer *i*, Δ*z*_*i*_ is the corresponding layer thickness, and *n* layer is the number of the vertical layers in PMCAMx-SR. The extinction coefficient depends on the wavelength λ, and it is given for each layer *i* by
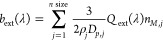
2where *Q*_ext_(λ) is the extinction efficiency, which depends on λ,^41^*j* is the aerosol size bin used in PMCAMx-SR, and it can take values from 1 to 10, *n*_*M*,*j*_ is the total concentration of all aerosol species for aerosol size bin *j*, and ρ_*j*_ and *D*_*p*,*j*_ are the density and the diameter of the particles, respectively. The density ρ_*j*_ is given by
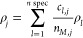
3where *c*_*l*,*j*_ is the concentration of each aerosol species for aerosol size bin *j*, and ρ_*l*_ is the density of each particle component. The assumed densities for each component are given in Table S1. The total number of particle species predicted by PMCAMx-SR (*n* spec) is 11 (Table S1).

The absorption efficiency *Q*_abs_(λ) is the difference between the extinction efficiency *Q*_ext_(λ) and the scattering efficiency *Q*_scat_(λ)^41,42^ and is given by
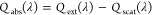
4The quantities of *Q*_ext_(λ) and *Q*_scat_(λ) are both given by Mie theory.^43^ For the calculation of *b*_scat_(λ) and *b*_abs_(λ), eq 2 is used, replacing *Q*_ext_(λ) with *Q*_scat_(λ) and *Q*_abs_(λ), respectively, while for the calculation of AOD_scat_, eq 1 is used, replacing *b*_ext,*i*_(λ) with *b*_scat,*i*_(λ).

The single scattering albedo (SSA) and the absorption AOD (AAOD), which also depend on λ, are given by
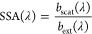
5
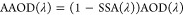
6The values of SSA(λ), AOD(λ), and AAOD(λ) are calculated for the total atmospheric column (and thus for all the simulated layers from PMCAMx-SR). In order to calculate the SSA for the total atmospheric column, we used the methodology of Péré et al.,^44^ weighing the SSA of each layer by the extinction coefficient of each layer.

For the complex refractive indices, we used the values used by Panagiotopoulou et al.^45^ for all the PM components except BrC. The complex refractive index of BrC is calculated based on the results of Zhang et al.^46^ and depends on wavelength. The real part of the refractive index for all three components of BrC is assumed to be equal to this of OA, thus equal to 1.5. The imaginary part *k*_BrC_(λ) is calculated based on Liu et al.:^47^
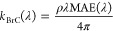
7where ΜΑΕ(λ) is the wavelength-dependent mass absorption efficiency of BrC:^48^
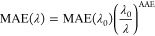
8where λ_0_, following Zhang et al.,^46^ is equal to 550 nm, and AAE is the absorption Ångström exponent. For the MAE(λ_0_), we used the value of 1 m^2^ g^–1^ for the iBrC and rBrC species, which is representative of biomass burning BrC.^49^ This value was also used by recent studies of Jo et al.^24^ and Zhang et al.^46^ For the phBrC, we used a value of MAE(λ_0_) equal to 0.19 m^2^ g^–1^ based on Zhang et al.^46^ and Nakayama et al.^50^ We also used the value of 5 for AAE (for the wavelength pair λ_0_ = 550 nm, λ = 440 nm), which is characteristic of biomass burning.^24,46,51^ With these above values, the imaginary part of the refractive index at 550 nm for the inert and reactive BrC is equal to 0.044 and for the photobleached BrC it is equal to 0.008. These are both consistent with the calculations of Zhang et al.^46^ We also assumed that the remaining OA species are only scattering. Assuming that the particulate matter is an internal mixture, the refractive index of the mixture is given by Pilinis.^52^

### 2.4 Method for the Evaluation of the Aerosol Optical Properties

A detailed comparison of PMCAMx predictions with MODIS AOD data has been performed by Panagiotopoulou et al.,^45^ so in this study we focus on PCMAMx-SR evaluation against AERONET^53,54^ data. We evaluated the predicted aerosol optical properties against data from the AERONET on an hourly basis. We used the AERONET version 3 level 2.0 products of AOD and extinction Ångström exponent (EAE) as well as inversion products for AAOD and SSA. Detailed descriptions of the instrumentation, calibration, methodology, data processing, and data quality assurance are provided by previous studies.^53−58^

We used specific criteria to focus on time periods and areas that were affected mainly by biomass burning. The first criterion used was that the hourly averaged concentration of bbOA in each grid cell examined had to be greater than the concentration of OA originating from other sources. The other criteria used ensured the absence of dust according to Chung et al.^26^ and to Bahadur et al.^59^ The extinction Ångström exponent (EAE) between 440 and 870 nm is given by
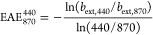
9where *b*_ext_ is the extinction coefficient at the two different wavelengths. We used values of EAE_870_^440^ > 1.0, which ensure the absence of dust, according to Chung et al.^26^ The scattering Ångström exponent (SAE) is defined similarly to the EAE, but for scattering:
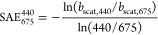
10where *b*_scat_ is the scattering coefficient at the two different wavelengths defined, 440 and 675 nm.

The absorption Ångström exponent (AAE) is defined in the same way as the EAE and SAE, but for absorption:
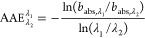
11where *b*_abs_ is the absorption coefficient at the two different wavelengths λ_1_ and λ_2_, respectively.
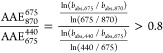
12We also used values of SAE_675_^440^ > 1.2 and eq 12 to ensure that the conditions are dust-free.^59^Equation 12 was suggested by Bahadur et al.^59^ by parameterizations for AAE concerning periods and sites that were not affected by dust, but only affected by carbonaceous aerosols.

### 2.5 Model Application

In the current study we applied the PMCAMx-SR model over Europe covering a region of 5400 × 5832 km^2^ described by a polar stereographic map projection using a 36 × 36 km^2^ horizontal resolution and 14 vertical layers that extend up to 7.5 km. We applied the PMCAM-SR chemical transport model during the 2012 summer PEGASOS campaign (June 5 to July 8, 2012).

#### 2.5.1 Brown Carbon Emissions from Biomass Burning

The wildfire emissions used by the PMCAMx-SR model, are based on the emissions provided by IS4FIRES by the Finnish Meteorological Institute.^60^ The estimated emissions of OA that originated from biomass burning during that period were 67% of the total OA emitted in the whole European domain. The average injection height is around 1 km, and only 2% of the fire emissions at this period are injected at a height above 2 km.

**Figure 1:**
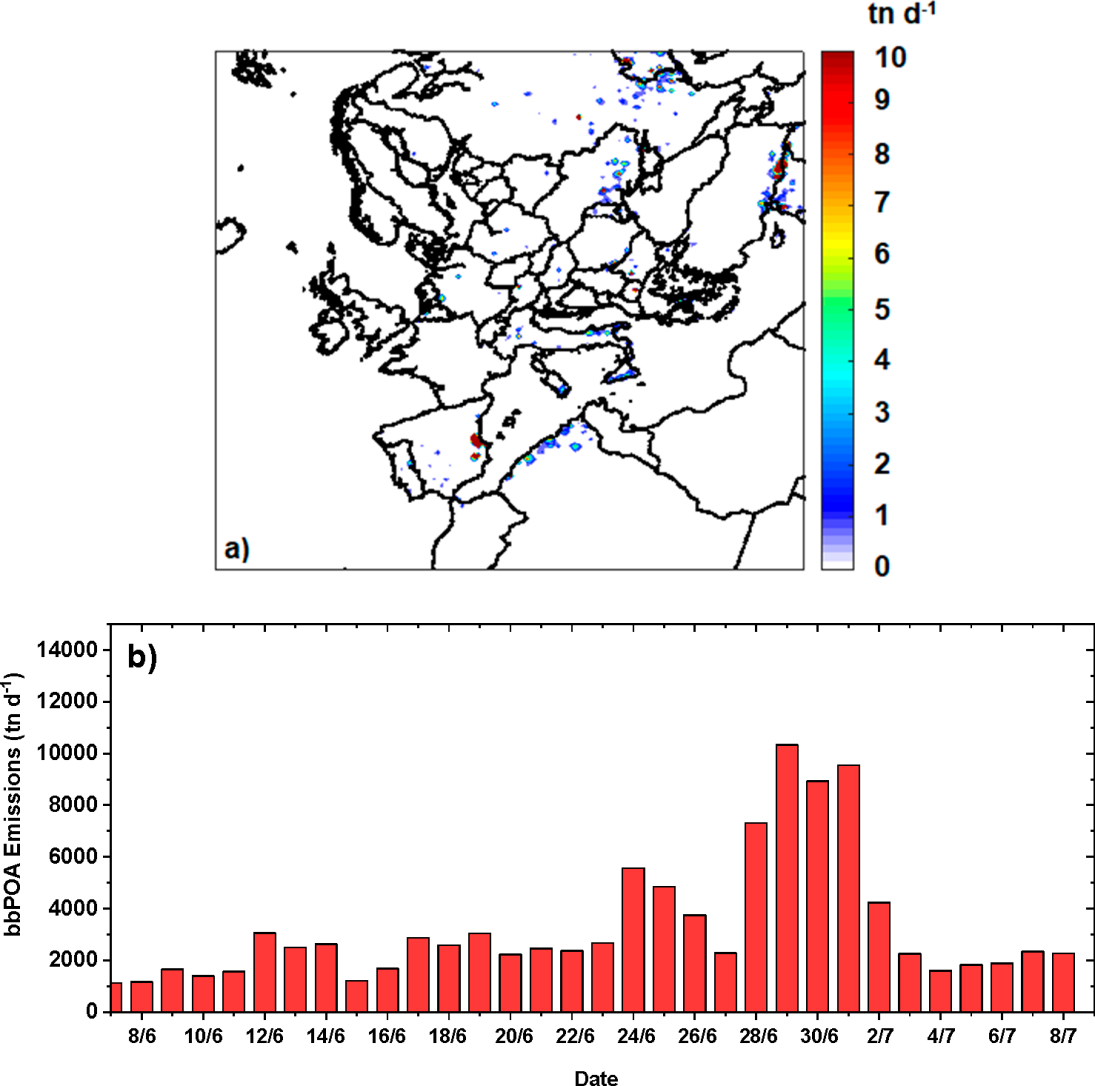
(a) Spatial and (b) temporal variations of the biomass burning organic aerosol emissions (including BrC) in tn d^–1^.

BrC is assumed to be part of the biomass burning organic aerosol (OA). The method of Zhang et al.^46^ is used to estimate the BrC emission rates, which are scaled to the OA and BC emissions from wildfires.^6^ Regarding this method, except the BC and OA emission rates, which are given from the wildfire emission inventory, we also used the MAE equal to 1 m^2^ g^–1^ assumed for BrC at 550 nm. 10% of these BrC emissions is assumed to be inert and the most persistent part of BrC, while the other 90% is assumed to be the reactive part.^5,61^ In total, average emissions of 170 tn d^–1^ of inert BrC and 1550 tn d^–1^ of reactive BrC were estimated. These BrC emissions are assumed to be part of the initial bbOA emissions, which were estimated to be 3130 tn d^–1^ on average. Figure 1a depicts the spatial distribution of these emissions during the simulated period, indicating major wildfires in Spain, in southern Italy, and at the borders of Turkey with Syria. The total bbOA emissions reached 10000 tonnes during June 29, when the major wildfire in Spain was taking place (Figure 1b).

#### 2.5.2 Other Inputs

All the meteorological fields used as inputs for the PMCAMx-SR model are provided by the Weather Research and Forecast Model (WRF).^62^ Biogenic emissions were estimated by the Model of Emissions of Gases and Aerosols from Nature v3 (MEGAN),^63^ marine emissions are based on a marine emission model,^64^ and anthropogenic emissions are based on the TNO emission inventory.^65^ The boundary conditions used are based on measured average concentrations in sites close to the boundaries of the domain, as in Fountoukis et al.,^36^ and are given in Table S2. The first 3 days of the simulation were not included in the analysis and were used as spin-up time.

## 3 Results and Discussions

### 3.1 Predicted Concentration Fields

**Figure 2:**
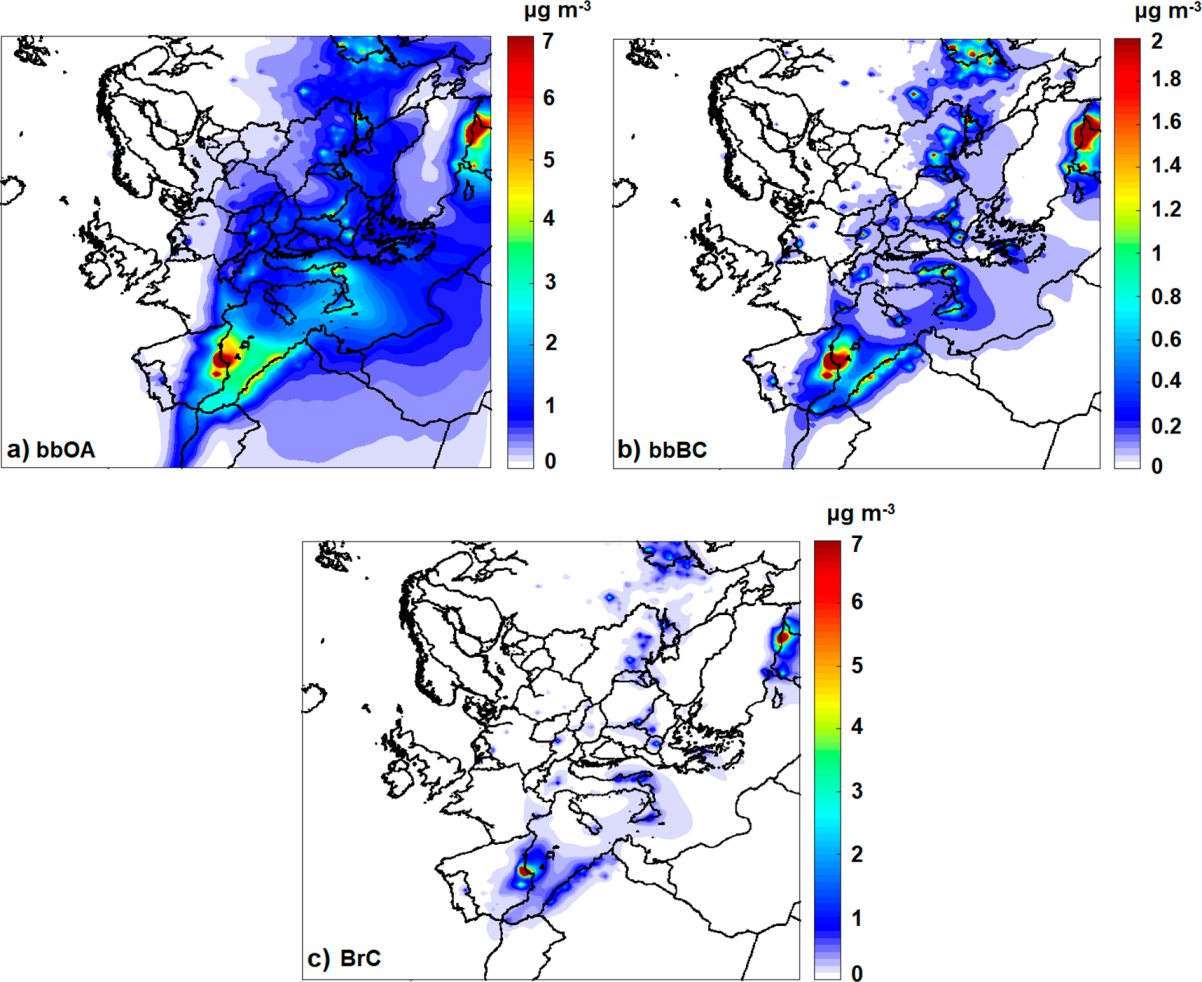
Predicted average ground level concentrations in μg m^–3^ of (a) bbOA (including BrC), (b) bbBC, and (c) BrC (iBrC+rBrC+phBrC).

PMCAMx-SR predicted ground-level concentrations of biomass-burning-related carbonaceous aerosols, averaged for the simulation period of (June 8 to July 8), are depicted in Figure 2.

Major fires were present near Valencia (Spain), in south Italy, and at the borders of Turkey with Syria (Figure 2a). The concentrations of bbOA, which are the sum of bbPOA and bbSOA, were more than 20 μg m^–3^ near the fire events. The predicted black carbon concentrations originating from wildfires (bbBC) decreased rapidly away from their sources, while bbOA levels were reduced more gradually due to the production of bbSOA (Figure 2b). The predicted average concentrations of BrC (Figure 2c) were almost 10 μg m^–3^ near the big fire events in Spain and Turkey-Syria.

**Figure 3:**
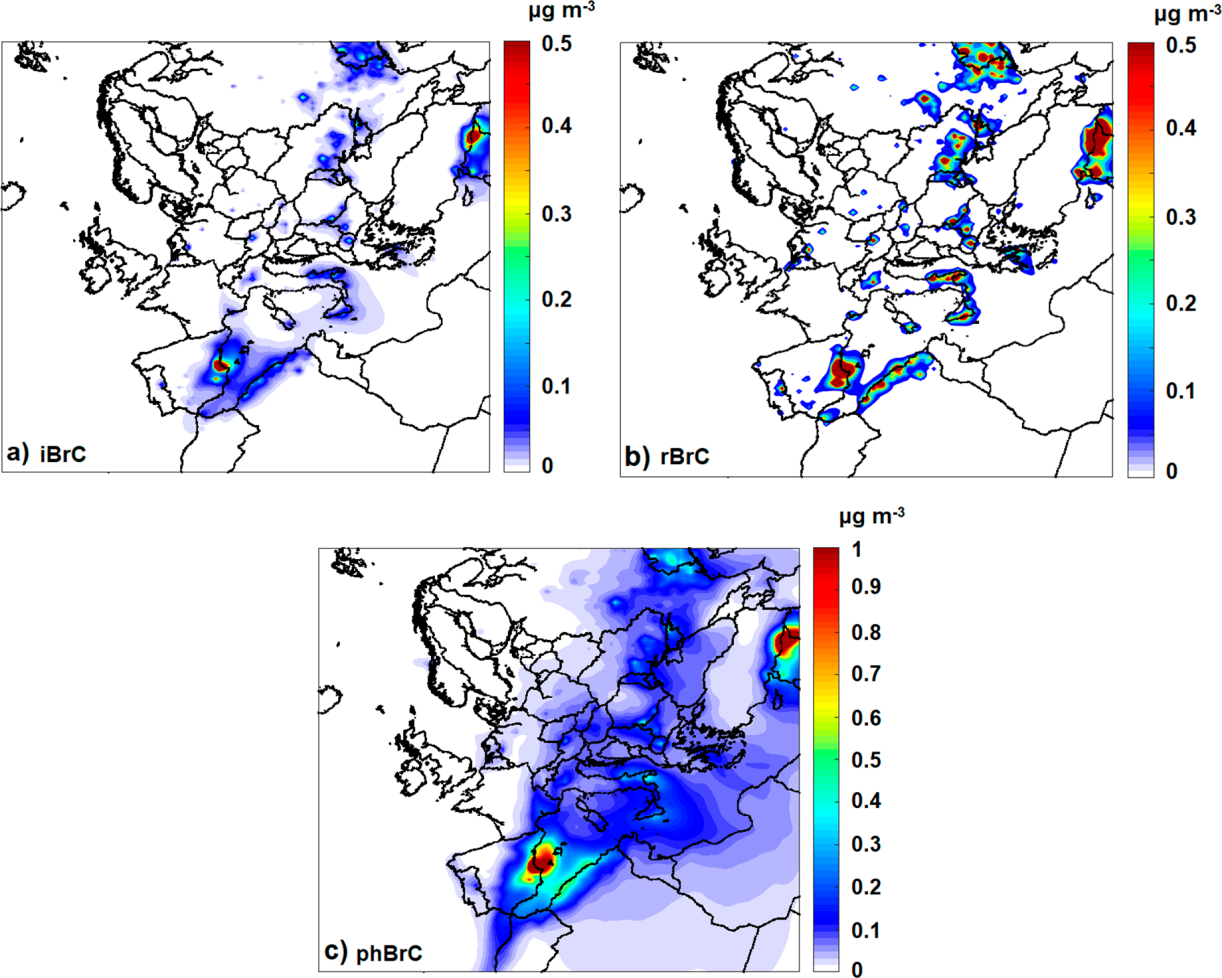
Predicted average ground level concentrations in μg m^–3^ of (a) inert BrC (iBrC), (b) reactive BrC (rBrC), and (c) photobleached BrC (phBrC).

The two main constituents of BrC, inert and photobleached BrC, had different concentration patterns across Europe. The inert BrC (Figure 3a) was higher close to the big fires and did not exceed on average the 1 μg m^–3^. This component of BrC is assumed to be chemically inert and to be removed only by wet and dry deposition.

The reactive BrC, even if it reacts to form photobleached BrC, is predicted to have higher concentrations than iBrC (Fig. 3b), exceeding 5 μg m^–3^ near the fires. The photobleached BrC average concentration varies from 2 μg m^–3^ near the fires to 0.1–0.2 μg m^–3^ in areas more than 1000 km away (Figures 3c and S1c). BrC concentration reached its ground level peak of 7 μg m^–3^ during June 30 in Barcelona (Spain), which is almost 300 km away from the Valencia wildfire (Figure S2a). During July 3 the wildfire started again, and on July 4 it started impacting Barcelona with BrC concentrations of 2 μg m^–3^. Even in Avignon (France), which is almost 800 km away from the Valencia wildfire, BrC concentrations reached, according to PMCAMx-SR, 1 μg m^–3^ during July 5 (Figure S2b). These results highlight the role of long-range transport and atmospheric circulation in the surface concentrations of BrC.

### 3.2 Predicted Aerosol Optical Properties

**Figure 4:**
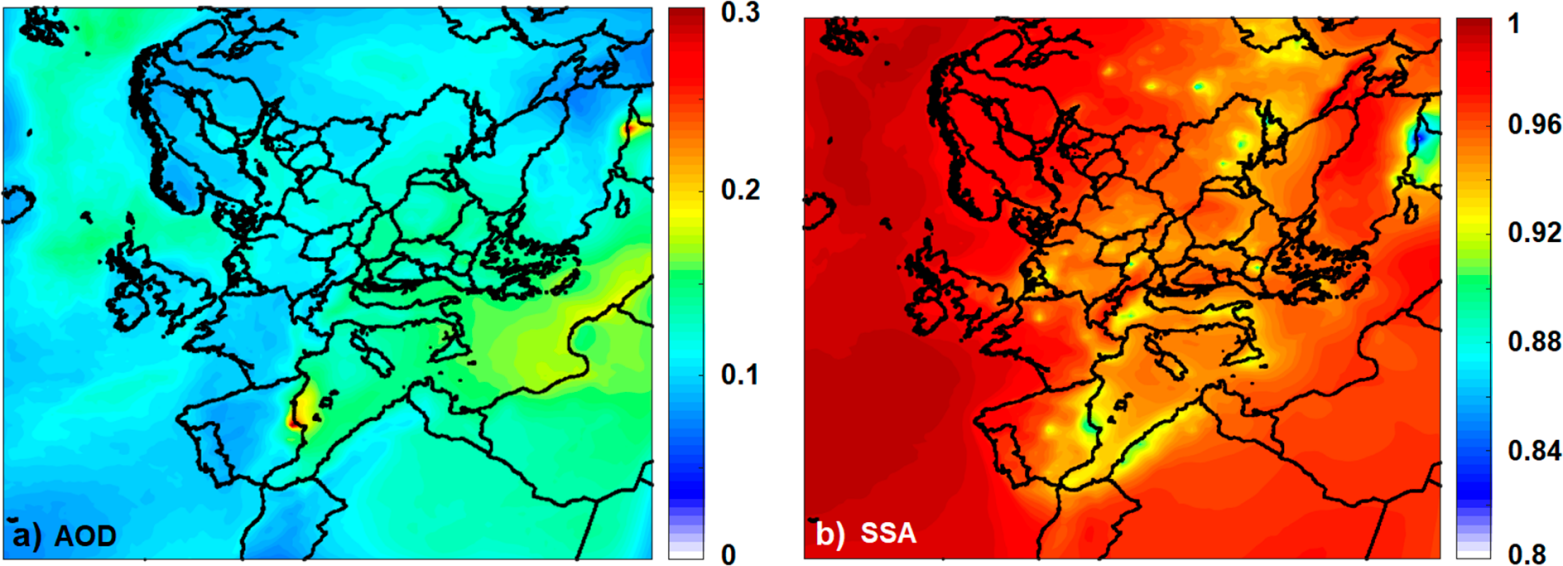
Predicted average (a) AOD at 550 nm and (b) SSA at 550 nm.

We examine first the average values of the total aerosol optical properties for the whole period, and then we focus on specific wildfire events. Predicted AOD has a maximum average value of 0.45 over the areas with high concentrations of PM originating from big wildfire events in Spain and at the borders of Turkey with Syria (Figure 4). AOD has a value of almost 0.2 over parts of the Mediterranean due to high sulfate concentrations from sources other than wildfires (Fig. 4a).

SSA on the other hand reached its minimum average value of 0.81 over the area of the wildfire at Turkey. SSA decreases as the particles become more absorbing (due to the existence of bbBC and BrC) near the fire. Predicted SSA was between 0.92 and 0.9 in areas that are affected by local anthropogenic BC sources like Madrid, Milan, and Paris, or had high bbBC and BrC concentrations from wildfires like fire events in Spain, south Italy, Ukraine, and Russia (Figure 4b). However, in these cases, the relatively low SSA is not associated with high AOD.

#### 3.2.1 Evaluation of the Predicted Optical Properties

We assume that the evaluation of the BrC predictions against AERONET measurements is valid, although there are limitations regarding the size distribution for the particles, the mixing state, and the maximum simulated height. Panagiotopoulou at al.^45^ explored the sensitivity of PMCAMx AOD predictions to size distribution and to mixing states and found that the modification is minor. To address the potential uncertainty introduced by not including layers at even higher altitudes (above 7.5 km), we quantified the effect of the aerosol at the higher levels of our simulation domain. We found that this contributed only 7% to the total AAOD. Therefore, we expect that at higher atmospheric levels, that impact will be much less.

**Figure 5:**
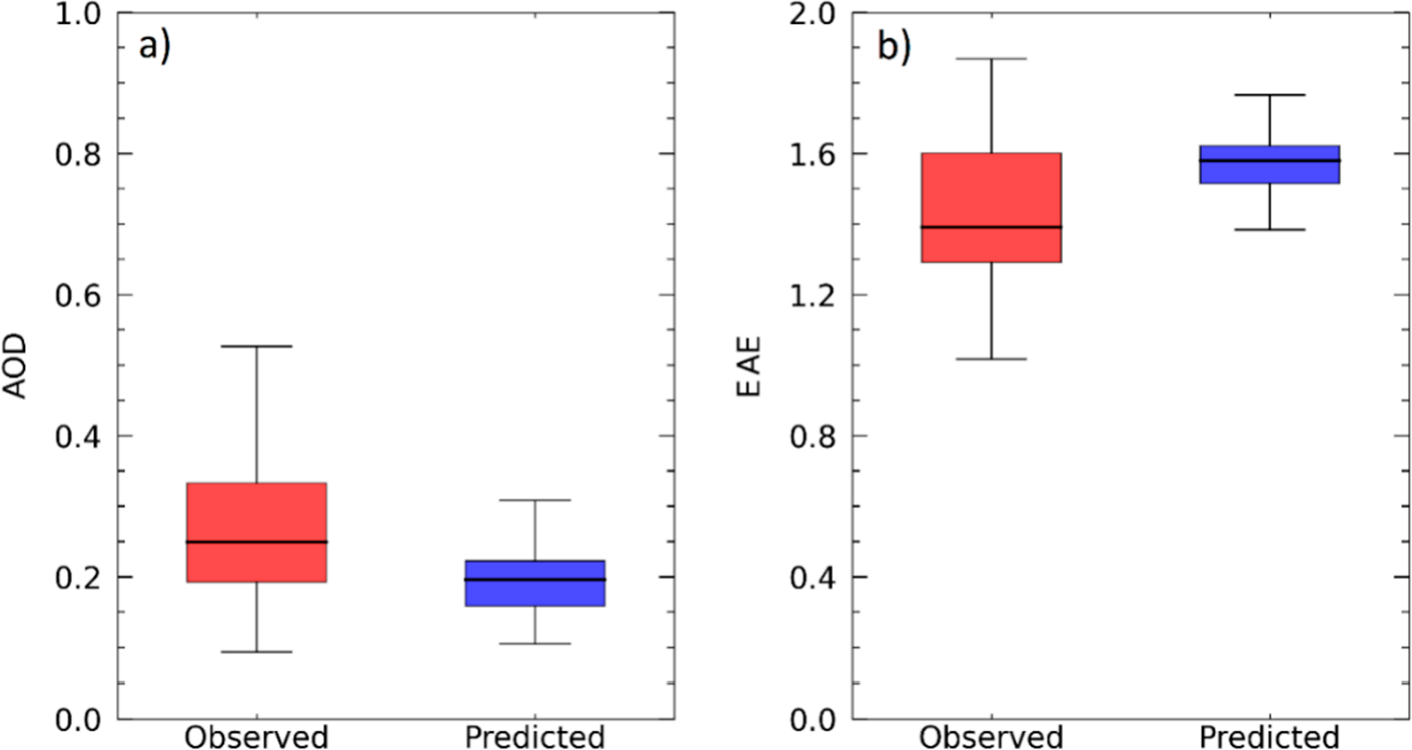
Predicted versus observed (219 points) for (a) AOD at 440 nm and (b) EAE_870_^440^.

We applied the following criteria, EAE_870_^440^ > 1.0, SAE_675_^440^ > 1.2 and eq 12 for the evaluation of the AOD and the EAE_870_^440^. The total number of points that meet the criteria described above is 219, and the AERONET stations used are given in Table S3. We used the fractional bias (*F*_bias_) and fractional error (*F*_error_) defined in Fountoukis et al.^36^ to evaluate the model. The performance of the model for AOD at 440 nm is quite good, with a *F*_bias_ of −0.25 and *F*_error_ of 0.40 and that for EAE_870_^440^ is even better, with *F*_bias_ of 0.1 and *F*_error_ of 0.15. The distributions of the predicted and observed (from AERONET) hourly AOD and EAE are shown in Figure 5.

We also used the AERONET inversion products in order to evaluate our model against SSA and AAOD at 440 nm. Following Wang at al.^66^ we used AAOD values greater than 0.01. The SSA and AAOD were evaluated against 74 available hourly observations across Europe (Table 1). The number of available data points is lower compared to AOD and EAE_870_^440^, because there are limited SSA values, which are used for the calculation of AAOD. The addition of predicted BrC has a small effect on the predictions of the SSA (Table 1). As a result, the *F*_bias_ and *F*_error_ for the two different cases are almost the same (*F*_bias_ = −0.02 and *F*_error_ = 0.04). However, the addition of BrC improves the performance of the model for AAOD. The *F*_bias_ improved from −0.3 to −0.24, and the *F*_error_ reduced from 0.51 to 0.48 (Table 1).

**Table 1:** PMCAMx-SR Evaluation Metrics for EAE_870_^440^, AOD, SSA, and AAOD at 440 nm

	without BrC		with BrC	
	*F*_bias_	*F*_error_	*F*_bias_	*F*_error_
EAE_870_^440^	0.11	0.16	0.10	0.15
AOD	–0.26	0.41	–0.25	0.40
SSA	–0.019	0.044	–0.02	0.046
AAOD	–0.3	0.51	–0.24	0.48

#### 3.2.2 Impact of BrC on the Predicted Aerosol Optical Properties at Different Wavelengths

BrC is more absorbing at shorter wavelengths, so it is interesting to examine how much the aerosol optical properties are influenced at different wavelengths. We performed an extra simulation of PMCAMx-SR, neglecting BrC. Then through zero-out analysis, we calculated the impact of BrC on the aerosol optical properties.

**Figure 6:**
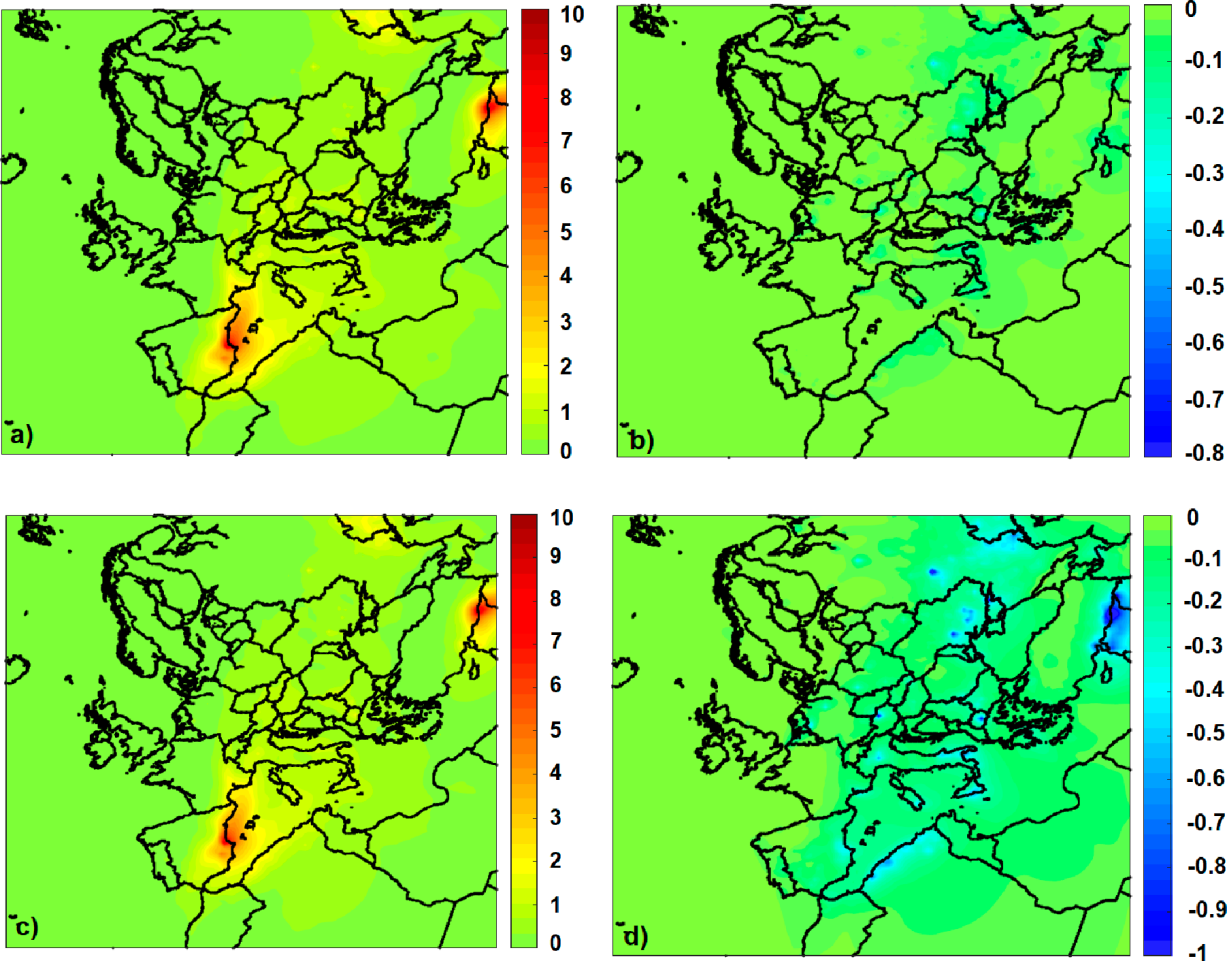
Predicted average percentage impact of BrC on (a) AOD at 550 nm, (b) SSA at 550 nm, (c) AOD at 440 nm, and (d) SSA at 440 nm.

For the case of the wildfire of Valencia, the AOD at the areas close to the fire increased by around 10% due to the presence of BrC. At areas 800–1000 km away from the wildfires (such as Avignon in France) it increased about 2% (Figure 6a,c). The increase is almost the same at 550 and 440 nm because the extinction coefficient of BrC does not change significantly (2–3%) in this wavelength range.

The impact of BrC on SSA is low compared to that on AOD, but the effect at 440 nm is almost 2 to 3 times higher than that at 550 nm. The effect of BrC on SSA at 550 nm is low at −0.5% on average (Figure 6b) and mostly lower than 0.2–0.3%. At 440 nm (Figure 6d), the existence of BrC decreases SSA by −1.5% in areas affected by fire smoke (for example, at the borders of Turkey with Syria).

**Figure 7:**
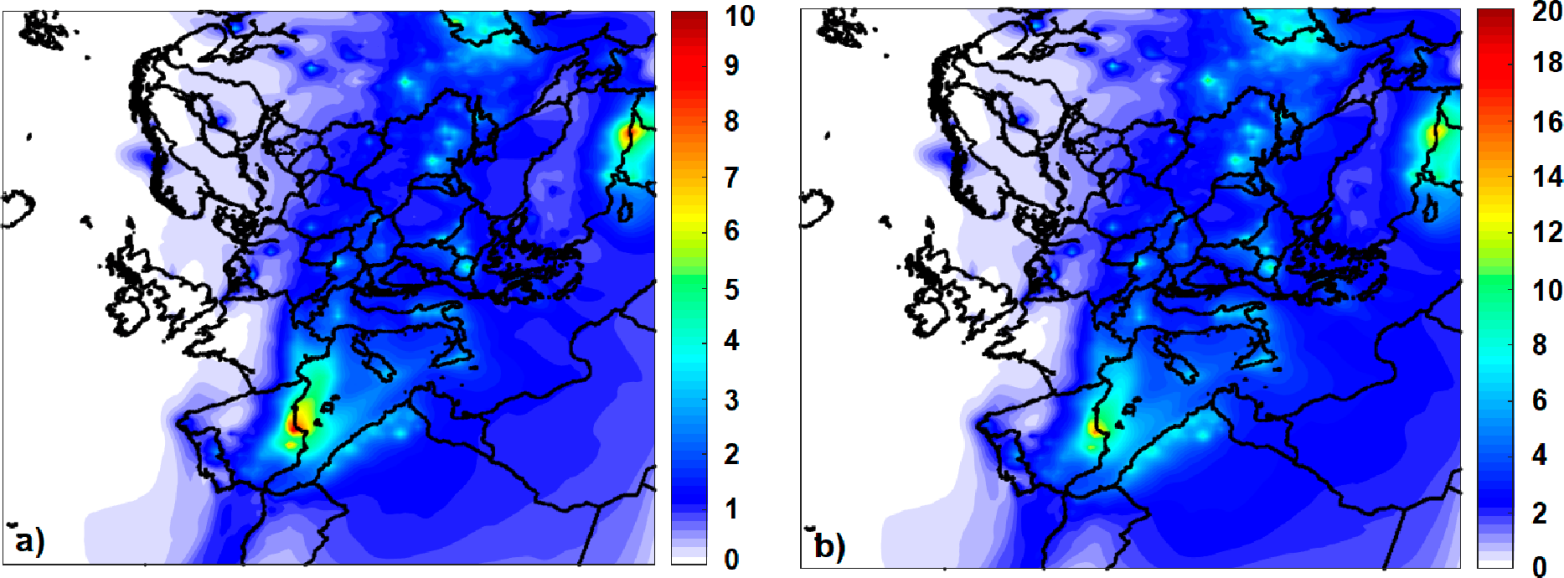
Predicted average percentage impact of BrC on (a) AAOD at 550 nm and (b) AAOD at 440 nm.

At 550 nm (Figure 7a), BrC causes an increase of the AAOD by 10% near major fire events and around 5–6% in areas 800–1000 km far from the fire which were affected by the smoke. At 440 nm (Figure 7b), the influence of BrC is stronger, 16% in areas near the major fire events (for example, near Valencia) due to the combined effect of BrC on both AOD and SSA (eq 7).

#### 3.2.3 Impact of Photobleaching of BrC on the Predicted Aerosol Optical Properties

As BrC-containing particles undergo photobleaching, they become less absorbing, and this affects the simulated AOD and SSA. We performed an extra simulation, neglecting photobleaching of BrC. Then through zero-out analysis, we calculated the effect of the photobleaching of BrC on the aerosol optical properties.

**Figure 8:**
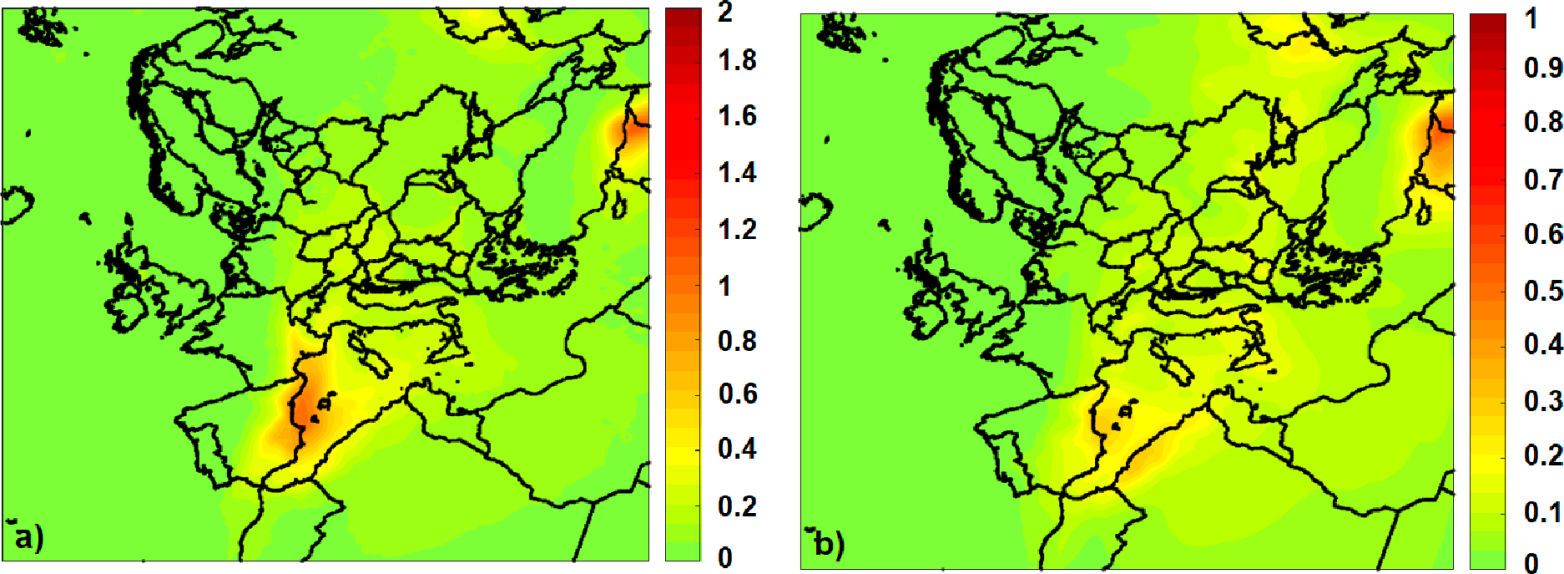
Predicted average percentage impact of photobleaching of BrC on (a) AOD and (b) SSA at 550 nm.

Photobleaching of BrC causes an increase in both AOD and SSA at 550 nm (Figure 8). AOD increased by 1–1.5% near the big fire events, and SSA increased around 0.6% due to the increase of the phBrC concentrations.

We have assumed that phBrC has a lower density than the inert and reactive BrC. The corresponding densities are reported in Table S1. As a result, photobleaching reduces the density of the organic aerosol in the model, and this leads to an increase of the AOD based on eqs 1 and 2. Far from wildfire events (800–1000 km) AOD increased by 0.5% and SSA by 0.3%.

We also performed an extra simulation neglecting bbBC and another one neglecting the biomass burning process. These results are presented in the Supporting Information.

### 3.3 Case Study: June 29

During June 29 the maximum daily values of the influence of the biomass burning to the aerosol optical properties were predicted due to the major wildfire near Valencia. Biomass burning caused an increase near the major wildfire of Valencia to the AOD by 96% both at 550 and at 440 nm (Table 2). It also caused a decrease to the SSA of 42.6% at 550 nm and 48.5% at 440 nm. The impact to the AAOD is high at 99% at both 550 and 440 nm (Table 2).

**Table 2:** Maximum Impact (%) of Different Cases on Optical Properties at 550 nm and at 440 nm on June 29

	impact on AOD (%)		impact on AAOD (%)		impact on SSA (%)	
	550 nm	440 nm	550 nm	440 nm	550 nm	440 nm
BrC	14.6	14.6	11.7	17.9	–0.7	–3.0
photobleaching	2.0	1.6	–5.0	–10.4	1.6	2.6
bbBC	19.2	20.3	87.9	78.9	–37.0	–33.2
biomass burning	96.3	96.4	99.3	99.3	–42.6	–48.5

BrC is expected to have a lower impact on AOD and SSA compared to bbBC. Near the major wildfire of Valencia, BrC contributed 14.6% to AOD at both 440 and 550 nm, of which 11.4% is due to the increase in scattering and the rest 3.2% due to the increase in absorption. The impact of bbBC on AOD was a little bit higher than that of BrC (19% and 20% at 550 and 440 nm, respectively; Table 2). The addition of BrC in the mixture causes more or less the same increase in AOD as the addition of bbBC, because the higher volume fraction of BrC compensates for the lower values of its refractive index. The decrease in SSA caused by bbBC is almost 10 times more compared to that caused by BrC at 440 nm (Table 2).

On the other hand, bbBC causes a higher increase in AAOD at 550 and 440 nm because bbBC is more absorbing than BrC, as indicated in Table 2. Biomass burning BC causes an increase in AAOD that is 7.5 times higher than that of BrC at 550 nm and 4 times higher at 440 nm (Table 2).

In addition, following ref (24), we performed a sensitivity analysis, changing AAE from 5 to 5.48 and then to 6.19. We applied the above two values in eq 8 and then calculated again through eq 7 the imaginary part of BrC. Using these values of the imaginary part of BrC, we performed two new simulations for the June 29 and we found that the total optical depth of absorption of the particles at 440 nm, decreased on average 0.4% using AAE = 5.48 (compared to AAE = 5) and 1% using AAE = 6.19, respectively.

Summarizing the results of this study, there are some new features which allow better simulation of BrC. The addition of three new species BrC, allow to give different values for their parameters (densities, MAE and AAE). For future studies, the assumptions for the optical properties of BrC have to be revisited (like the MAE and AAE) regarding the new measured parameters that may be available during biomass burning events. Additional measurements are clearly needed to better constrain these values and determine their variability in wildfires on different continents. In addition, the use of a recently reported photobleaching constant relevant to field measurements in the Mediterranean region is also a new feature. The assumption of newly formed phBrC species allows us to predict BrC concentrations produced by photobleaching. This is different compared to previous studies which assumed that the photobleaching directly affects the refractive index of BrC. The lifetime of BrC in the atmosphere calculated by Wong et al.^18^ is in line with previous studies conducted in northwestern U.S.^5^ and the Amazon,^66^ indicating that this constant can be also used as a first approximation at least in other regions. Further field data from different geographical regions are necessary for assessing the estimated BrC atmospheric lifetime and improving predictions of global BrC impacts. The use of an OH-independent constant for photobleaching of BrC is clearly a zeroth-order approximation that allows us to estimate the magnitude of the corresponding effects of photobleaching in this specific period. An extension of the model with an OH-dependent parametrization will be used in future work. Moreover, another limitation is the top layer of 7.5 km used in this application. For at least the simulation period, our choice of the top of the modeling domain does not have a significant effect on the simulation results due to the average injection height of wildfire emissions. However, in other cases, this height may be too low and has to be incorporated into future applications of the PMCAMx-SR model.

Finally, based on these results, our first estimates of BrC impact on AAOD seem promising, and together with future studies, they may shed light on the role of BrC in the optical properties of atmospheric aerosol.

## Supporting Information Available

Table with the description of the applied densities of each simulated species by the model; Table with the boundary conditions used in the simulations; Table with the description on the AERONET sites used in this study; Figures of the predicted bbOA concentrations during different simulation days; Figures of the predicted ground level BrC components concentrations, for different locations, during fire event in Valencia; Paragraph describing the impact of bbBC and biomass burning on the predicted aerosol properties (PDF)
