# Supplementary material for: Predicted Concentrations and Optical Properties of Brown Carbon from Biomass Burning over Europe
Source: ACS EST Air. 2024 May 28;1(8):897–908. doi: 10.1021/acsestair.4c00032 (PMC11474818; doi:10.1021/acsestair.4c00032)
Supplement: Supplementary file 1 — ea4c00032_si_001.pdf [file ea4c00032_si_001.pdf]

Supporting Information

Predicted Concentrations and Optical Properties of Brown Carbon from Biomass Burning over Europe

Ksakousti Skyllakou[a], Marios-Bruno Korras-Carraca[a,c], Christos Matsoukas[b], Nikos Hatzianastassiou[c], Spyros N. Pandis[a,d*], Athanasios Nenes[a,e**]

[a]Institute of Chemical Engineering Sciences, ICEHT/FORTH, Patras 26504, Greece

[b]Department of Environment, University of the Aegean, Mytilene 81100, Greece

[c]Laboratory of Meteorology and Climatology, Department of Physics, University of Ioannina, Ioannina 45110, Greece

[d]Department of Chemical Engineering, University of Patras, Patras 26504, Greece

[e]Laboratory of Atmospheric Processes and their Impacts, École Polytechnique Fédérale de Lausanne 1015, Switzerland

*Email: spyros@chemeng.upatras.gr

**Email: athanasios.nenes@epfl.ch

Table S1: Densities used for each PM component by the algorithm for the calculation of the aerosol optical properties.

**PM components**	**Density (g cm^{-3})**
BC	2.0
Non absorbing OA (aSOA+bSOA+LRT+fPOA+bbOA)	1.0
Non absorbing BrC (phBrC)	1.0
Absorbing BrC (iBrC+rBrC)	1.5
Sulfate	1.5
Nitrate	1.5
Ammonium	1.5
Sodium	2.0
Chloride	2.0
Crustal Material	3.0
Water	1.0

Table S2: Aerosol concentrations (μg m^{-3}) at the boundaries of the domain.

	Boundaries			
Species	Top	Bottom	Left	Right
OA	1	0.5	0.5	1
Sulfate	1	1	1	1
Ammonium	0.37	0.37	0.37	0.37
Nitrate	0.1	0.02	0.01	0.1
Sodium	0.001	0.005	0.09	0.03
Chloride	0.002	0.01	0.1	0.05

Table S3: AERONET stations used in this study.

Site name	Country	Latitude	Longitude	Altitude (m ASL)
Avignon	France	43.93275	4.87807	32
Toulouse	France	43.57472	1.37389	150
Barcelona	Spain	41.38925	2.11206	125
Lampedusa	Italy	35.51667	12.63167	45
Minsk	Belarus	53.92000	27.60100	235
Kyiv	Ukraine	50.36361	30.49667	200
Moldova	Moldova	47.00080	28.81560	101
IMS-METU-Erdemli	Turkey	36.565	34.255	3

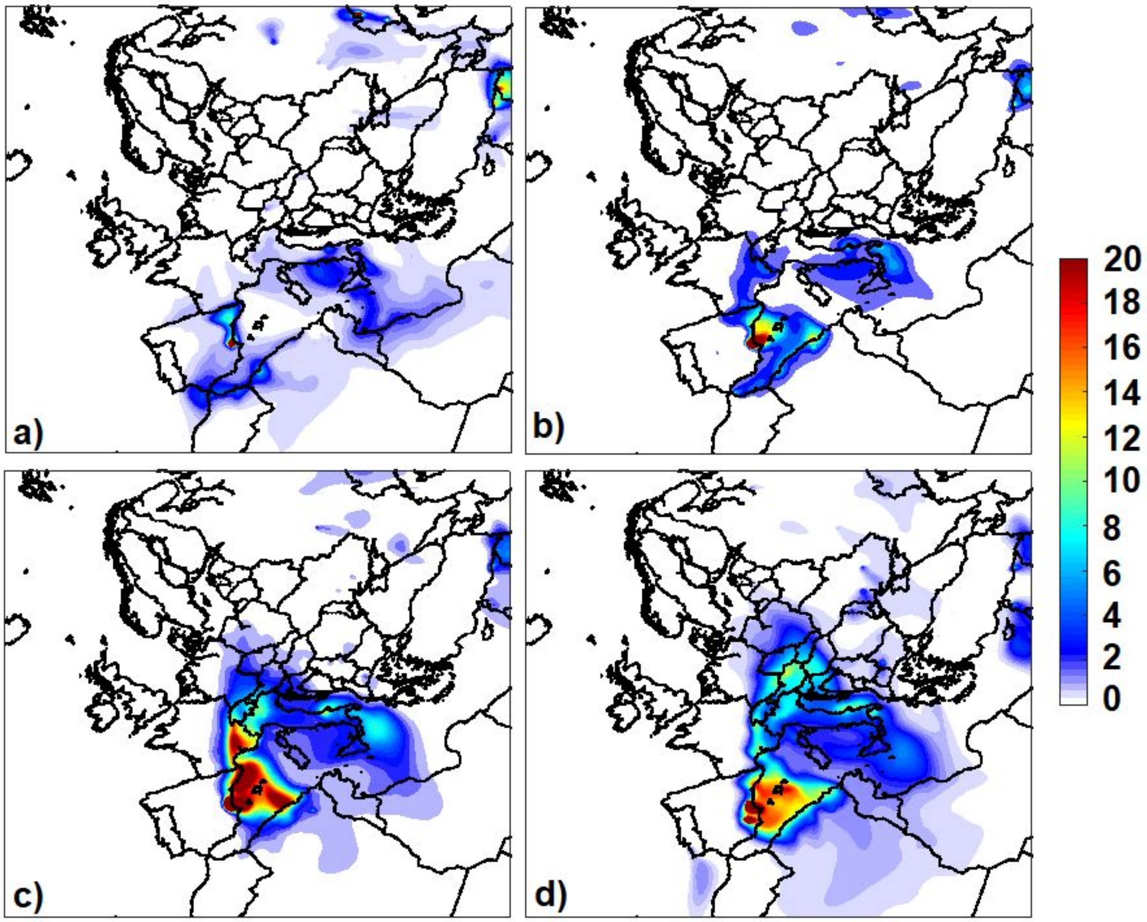

Figure S1: Predicted ground level bbOA concentrations in μg m^{-3}, during different simulation days: a) June 28, b) June 29, c) June 30, d) July 1.

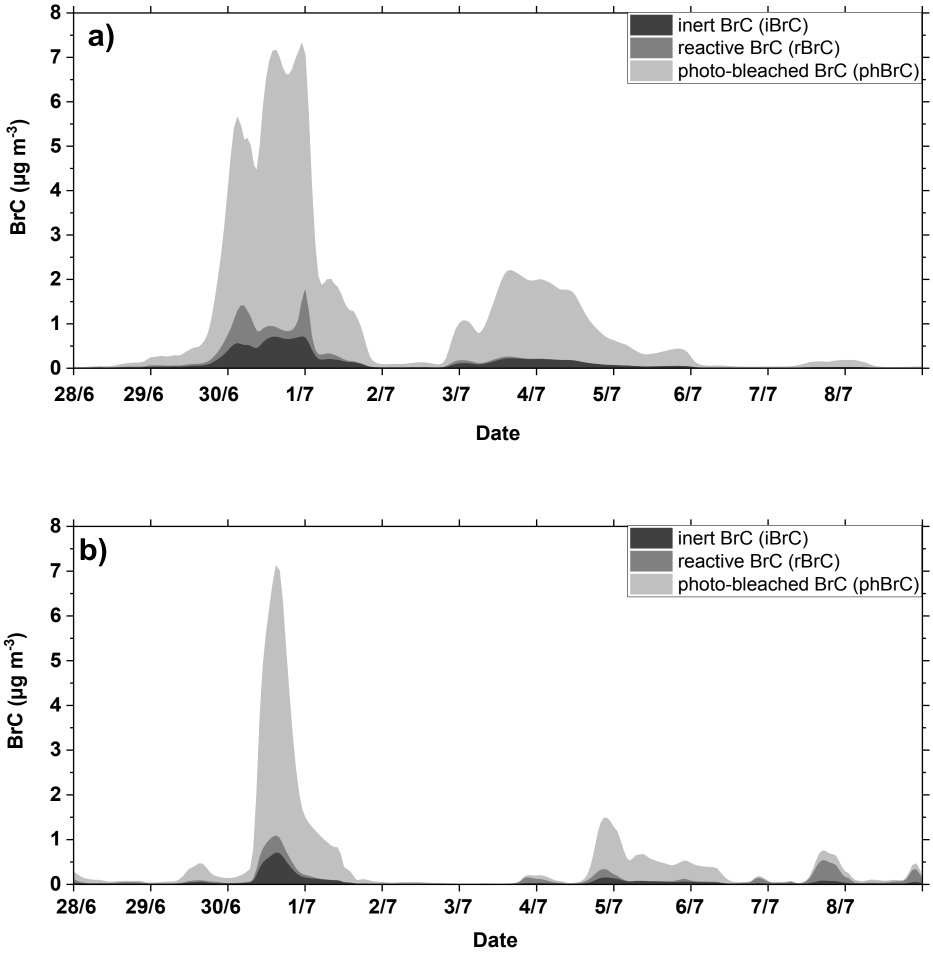

Figure S2: Predicted ground level main BrC components concentrations in µg m^{-3}, for different locations, during fire event in Valencia: a) Barcelona, Spain (approximately 300 km away from the fire), b) Avignon, France (approximately 800 km away from the fire).

Impact of bbBC and biomass burning on the predicted aerosol optical properties

We performed an extra simulation neglecting bbBC and another one neglecting the biomass burning process. This was done because biomass burning emissions include also other components (like dust) which are scattering rather than absorbing. There is strong evidence that the turbulence caused by the intense local heating of wildfires leads to the emissions of dust which is found then in smoke plumes (Alves et al., 2010; Wagner et al., 2018). Then through zero-out analysis we calculated the impact of bbBC and the impact of biomass burning on the optical properties at 550 nm.

bbBC significantly affects the predicted AOD at 550 nm, by causing an increase of almost 12% near the fire events (Fig. S3a) and almost 2-3% in areas far from the fires. On the other hand, bbBC causes a decrease in SSA by 19% in the major fire event at the borders of Syria with Turkey, and a decrease of about 7% in the big fire event near Valencia (Fig. S3b).

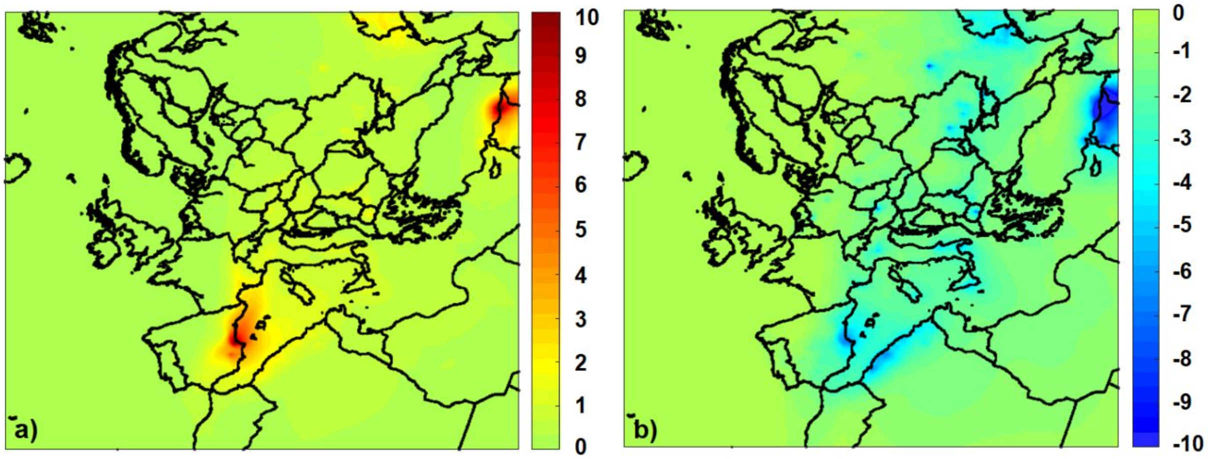

Figure S3: Predicted average percentage impact of bbBC on: a) AOD and b) SSA at 550 nm.

In total, biomass burning impacts much more the AOD compared to bbBC. This is expected because OA, BrC, and other PM components are also emitted during a fire. On the other hand, the change of SSA due to biomass burning is comparable to the corresponding change due to bbBC. For example, biomass burning causes an increase to the AOD at 550 nm (Fig. S4a) by almost 70% near the big fire events, and a decrease to the SSA at 550 nm of 20% (Fig. S4b). Far from wildfires (800-1000 km) AOD increases by 20% due to biomass burning and SSA decreases by 3-4%.

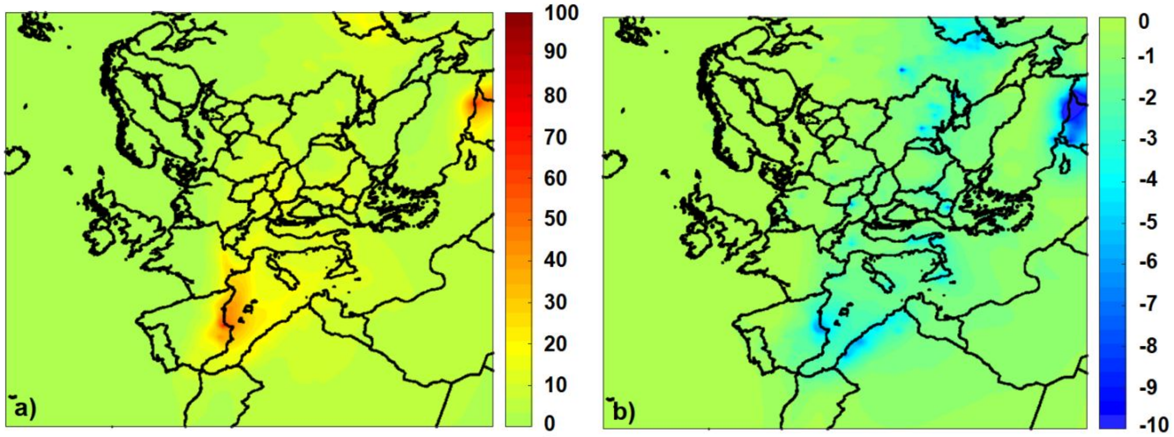

Figure S4: Predicted average percentage impact of biomass burning on: a) AOD and b) SSA at 550 nm.

References

Alves, C.A.; Gonçalves, C.; Pio, C.A.; Mirante, F.; Caseiro, A.; Tarelho, L.; Freitas, M.C.; Viegas, D.X. Smoke emissions from biomass burning in a Mediterranean shrubland, Atm. Environ., 44, 3024-3033, 2010.

Wagner, R., Jähn, M., and Schepanski, K. Wildfires as a source of airborne mineral dust-revisiting a conceptual model using large-eddy simulation (LES), Atmos. Chem. Phys., 18, 11863–11884, 2018.
